# Floral Color Variation in *Drosera cistiflora* Is Associated With Switches in Beetle Pollinator Assemblages

**DOI:** 10.3389/fpls.2020.606259

**Published:** 2020-11-17

**Authors:** Steven D. Johnson, Caitlin G. von Witt, Bruce Anderson

**Affiliations:** ^1^Centre for Functional Biodiversity, School of Life Sciences, University of KwaZulu-Natal, Pietermaritzburg, South Africa; ^2^Department of Botany and Zoology, Stellenbosch University, Stellenbosch, South Africa

**Keywords:** ecotypes, geographical races, local adaptation, hopliine beetle, phenotypic plasticity, pollination, pollen rewards, speciation

## Abstract

Floral color shifts are thought to be one of the most common evolutionary transitions in plants, and pollinators are often proposed as important selective agents driving these transitions. However, shifts in flower color can also be related to neutral genetic processes or pleiotropy linked with selection via other biotic agents or abiotic factors. Here we ask whether abiotic factors or pollinators provide the best explanation for divergence in flower color among populations of the sundew *Drosera cistiflora* s.l. (Droseraceae). This species complex in the Greater Cape Floristic Region contains at least five distinctive floral color forms. Abiotic factors do not appear to play a significant role in color determination, as the forms are not specific to a single soil or vegetation type, sometimes co-occur in the same habitat, and maintain their color traits in common-garden and soil switching experiments. Instead, we found strong associations between flower color and the composition of pollinator assemblages which are dominated by hopliine scarab beetles. Pollinator assemblages show geographical structuring, both within and among color forms. This makes it difficult to dissect the roles of geography versus floral traits in explaining pollinator assemblages, but strong pollinator partitioning among color forms at sites where they are sympatric indicates that pollinators may select strongly on color. These results suggest that beetle pollinators are a significant factor in the evolution of *D. cistiflora* s.l. flower color.

## Introduction

Pollinator-mediated selection on floral traits is considered a major driver of floral divergence (Grant and Grant, 1965; Fenster et al., 2004; Harder and Johnson, 2009; Schiestl and Johnson, 2013; Gervasi and Schiestl, 2017). It follows that spatial variation in pollinator assemblages may generate divergent selective pressures among plant populations, as proposed by the Grant-Stebbins pollinator-shift model (Grant and Grant, 1965; Stebbins, 1970; Johnson, 2006; Kay and Sargent, 2009; Van der Niet et al., 2014).

Convergent evolution of flower color is well documented for guilds of plants pollinated by the same pollinator functional group (Fenster et al., 2004) and this provides one line of evidence for pollinator-mediated selection on floral color evolution. Another is divergence in flower color among closely related plants that have pollinators differing in color preference. Indeed, there is now some compelling evidence for this process, albeit limited to few studies (Meléndez-Ackerman, 1997; Schemske and Bradshaw Jr. 1999; Irwin and Strauss, 2005; Hopkins and Rausher, 2012; Streinzer et al., 2019). Array experiments involving *Mimulus* species (Phrymaceae) and their hybrids, for example, have confirmed flower color discrimination by both hummingbirds and bees, and further linked pollinator preferences to specific gene regions associated with petal pigmentation and nectar volume (Schemske and Bradshaw Jr. 1999). By using arrays of both model and reciprocally translocated flowers, Newman et al. (2012) attributed geographical variations in flower color of the orchid *Disa ferruginea* to pollinator color preferences shaped by positive associative conditioning in local communities. New evidence also suggests that elevational segregation of flower color in *Anemone pavonina* (Ranunculaceae) may be linked to the relative importance for pollination of glaphyrid beetles versus other insects along environmental gradients (Streinzer et al., 2019). In addition, numerous experimental studies with flower-visiting animals have revealed strong innate color preferences as well as ability to develop color preferences through associative conditioning (Lunau et al., 1996; Weiss, 1997; Pohl et al., 2008; Ings et al., 2009).

Although these studies suggest that pollinators can be very important selective agents in floral color transitions, and even the main driving force behind these shifts, no study has demonstrated unequivocally that different assemblages of pollinators are the agents of selection behind geographical floral color shifts (Rausher, 2008). Several other hypotheses for color shifts have been proposed (Narbona et al., 2018), including nonadaptive evolution as a result of genetic drift (Wright, 1943) or the (indirect) consequence of pleiotropic effects of genes relating to physiological or vegetative adaptation to environmental conditions (Rausher and Fry, 1993; Levin and Brack, 1995; Warren and Mackenzie, 2001; Armbruster, 2002; Strauss and Whittall, 2006; Arista et al., 2013). For example, the anthocyanin pigment may confer abiotic stress tolerance to seedlings, and, since its presence in seedlings can also determine flower color (Bowman, 1987; Strauss and Whittall, 2006), floral color transitions may be maintained through selection on seedling traits. In *Acer* (Sapindaceae) red and purple flowers evolved in lineages where anthocyanins are present in leaves, while pale-green or yellow flowers evolved in lineages without anthocyanins in leaves (Armbruster, 2002). Alternatively, flower color genes may have pleiotropic effects on water use physiology, which in turn may result in geographical structuring of flower colors, as proposed for *Linanthus parryae* (Polemoniaceae) by Schemske and Bierzychudek (2001). Floral color divergence may also be maintained through the pleiotropic effects of flower color genes on herbivory (Irwin et al., 2003) and seed predation (Carlson and Holsinger, 2010, 2013). Lastly, floral color shifts may represent plastic responses to differing edaphic conditions such as geographical soil mosaics, as determined by variation in physical and/or chemical components of the soil (Ito et al., 2009). A well-known example is *Hydrangea macrophylla* (Hydrangeaceae), which can vary from blue to pink depending on soil pH (Ito et al., 2009; Schreiber et al., 2010). It is clear, therefore, that studies of flower color evolution should include an integrated consideration of biotic and abiotic factors that may explain transitions between flower colors (Herrera, 1996; Galen, 1999a,b; Ellis and Johnson, 2009).

*Drosera cistiflora* s.l. is a perennial, pollinator-dependent, insectivorous plant species complex endemic to the Fynbos Biome in the Greater Cape Floristic Region of South Africa. Plants produce inflorescences with 1–5 large flowers (30–55 mm in width) which open consecutively (usually only one flower is presented at a time) and are hermaphroditic, actinomorphic and bowl-shaped. There is a spectacular diversity of floral color forms in the complex, ranging through pink, purple, red, white and yellow (von Witt, 2019). Though currently recognized as a single species, *D. cistiflora* may be best characterized as a species complex consisting of numerous forms at different stages of divergence. Some floral color forms can co-exist without producing visible intermediates and may represent emerging lineages with near-identical morphology (von Witt, 2019). The purple, red, and yellow flower colors are discrete, but there can be a gradient between pink- and white-flowered forms in some populations, while other populations are entirely white- or pink-flowered. The flowers of *D. cistiflora* s.l. are pollen-rewarding and devoid of nectar and discernible scent, making them particularly suitable for studies on the role of flower color in influencing pollinator attraction. Evidence suggests that *D. cistiflora* s.l. is pollinated primarily by hopliine beetles [Coleoptera: Scarabaeidae: Hopliini] (Goldblatt et al., 1998; Anderson, 2010; von Witt, 2019). All floral color forms are dependent on pollinators for seed production, which is moderately to highly pollen-limited (von Witt, 2019; von Witt et al., 2020).

This study explores two alternative hypotheses: i) that floral color variation in *D*. *cistiflora* s.l. is correlated with abiotic factors such as soils or ii) that it is associated with pollinator assemblages. If floral color divergence is a plastic response to soils, or if it is an evolved response to differences in components of the physical environment, then we predict that populations with the same flower color should occur in similar soil and vegetation types. In addition, plastic responses to soils should also be characterized by changes in flower color when plants are grown in different soils. If floral color variation is driven by pollinator-mediated selection, then we expect that populations with the same flower color should have similar suites of insect pollinators, and that populations with different flower colors should have different pollinator assemblages.

## Materials and Methods

### Study Sites

Study sites were chosen to represent extant populations of *Drosera cistiflora* s.l. (Supplementary Table S1). We studied 16 populations representing the following flower colors (*n* = populations): pink (4), purple (2), red (4), white (3), and yellow (3).

### Geographical Distribution of Floral Color Forms

To determine whether there is any geographical pattern in flower color distribution, we mapped all records of historical *D*. *cistiflora* s.l. populations in South Africa according to flower color wherever this was documented in the collector’s notes. Records were obtained from specimens housed in the Compton and Bolus Herbaria; data collected by the Custodians of Rare and Endangered Wildflowers (CREW) program, and field excursions carried out in our personal capacity. These were mapped using ARCVIEW GIS 3.2. Voucher specimens are housed in the Compton Herbarium.

We determined the spectral reflectance over the UV–visible range (300–700 nm) of a sample from 5 to 16 petals, each from a separate plant, in populations of each color form. Populations sampled (see Supplementary Table S1 for site details) were Darling 6 and 7 (pink-flowered form); Darling 2 and Durbanville (purple-flowered form); Darling 1, 2 and 3, and Darling-Yzerfontein (red-flowered form); Betty’s Bay, and Darling 4 and 5 (white-flowered form), and Piketberg 1, 2, and 3 (yellow-flowered form). We used an Ocean Optics (Dunedin, FL, United States) S2000 spectrophotometer and Ocean Optics DT-mini deuterium tungsten halogen light source (200–1,100 nm). We took reflectance readings from the outer section of the petals by placing the fiber optic reflection probe (UV/VIS 400 μm) at a 45° angle from the surface of the petal.

### Soil Types and Vegetation

To investigate the potential for edaphic specialization in *D*. *cistiflora* s.l. floral color forms, we determined the underlying geology and soil structure of populations of each floral color form by overlaying the 1:250,000 geology layer (2010) from the Western Cape Department of Agriculture onto point locality data of *D*. *cistiflora* s.l. populations with recorded flower colors (Supplementary Table S2).

A common-garden and soil switching experiment was conducted to test whether differences in soil chemistry and altered environmental conditions influenced expression of flower color. We allocated plants in bud, from each of the floral forms to different potting treatments: Plants were either planted in their soil of origin, or in soils taken from the sites of each of the other color forms, or in a soil from a site without *D. cistiflora*. We used three plants per treatment per color form, making up a sample of 18 plants per color form and 90 plants in total. These were then moved to a common site and observed for changes in flower color (Supplementary Figure S1). Controls consisted of plants potted in their original soil. Plants and soils were obtained from Darling 7 (granite and granodiorite soils supporting the pink-flowered form); Darling 2 (loam and sandy loam soils; purple-flowered form); Darling 3 (loam and sandy loam soils; red-flowered form); Darling 4 (granite and granodiorite soils; white-flowered form), and Piketberg 1 (grit and greywacke soils; yellow-flowered form). For the soil treatment from a site without *D. cistiflora* s.l., we used clay soils collected from The Towers Farm, Darling.

Plants were potted at the beginning of the flowering season in 2010 and observations made until flowering ceased at the end of each season, up until 2013. All experimental plants were kept in common environmental conditions at all times, and these common conditions were altered when the experiment was transferred from Darling to the Kirstenbosch National Botanical Garden Collections Nursery greenhouse of the South African National Biodiversity Institute after the flowering season in 2010. The plants were thus exposed to changes in soil temperature, light and moisture availability when they were removed from their native sites and also during the course of the experiment. Flower color was allocated to the pink, purple, red, white and yellow categories by the human eye.

Considering that plant communities share similar abiotic environmental conditions, community classification may act as a surrogate for overall abiotic factors to be compared between *D*. *cistiflora* s.l. populations. Thus, to assess whether populations of the same *D*. *cistiflora* s.l. floral color form are associated with similar plant communities, we established the vegetation type of each population by overlaying the 1:250,000 vegetation layer (Mucina and Rutherford, 2006) onto point locality data for extant populations of all *D*. *cistiflora* s.l. floral color forms.

### Pollinator Assemblages

To determine whether the flower color of a *D*. *cistiflora* s.l. population was associated with the pollinator community, flower visitors were observed in 16 populations of five floral color forms in 2009 and 2010 on sunny, windless days during periods of peak pollinator activity: 09h30–15h00. Each site measured approximately 50 × 50 m. Where possible, 250 flowers were randomly checked for the presence of pollinators. Where fewer than 250 flowers were present, all flowers in the population were checked (Supplementary Table S1) and observation numbers standardized across populations. The abundance and kinds of flower visitors were noted and at least one voucher specimen of each visitor was captured for identification. Insects that came into contact with floral reproductive parts were considered to be pollinators. Individual insects were killed by freezing and kept in separate vials to avoid pollen contamination. All insects were identified to family or subfamily, and genus and species where possible.

The potential importance of each insect species as a *D. cistiflora* s.l. pollen vector was calculated as the product of its relative abundance as a visitor to *D*. *cistiflora* s.l. flowers within a population and the average number of *D*. *cistiflora* s.l. pollen grains that it carried. Pollen grains were counted under a dissecting microscope for 1–12 (median = 5) individuals (in some cases fewer than five individuals were captured) of all observed insect visitor species and classified as *D*. *cistiflora* s.l. pollen or “other.” Pollen grains were identified by comparison with a reference set of microscope slide preparations of pollen grains made from *D*. *cistiflora* s.l. and co-occurring plants at all study sites. Relative pollinator importance (RPI) was calculated as the percentage contribution of each pollinator to the overall pollinator importance for each *D*. *cistiflora* s.l. floral color form.

If pollinators discriminate among floral color forms or if pollinators are geographically associated with particular flower colors, then we would expect that populations of the same flower color will be visited by similar assemblages of insects. This was tested with non-metric multidimensional scaling (NMDS) plots of the Bray-Curtis index of pollinator species composition for *D*. *cistiflora* s.l. floral color forms. Three analyses were performed, one using relative pollinator abundances in each *D*. *cistiflora* s.l. population, another using an estimate of the importance of each pollinator species (as the product of relative abundance and average pollen loads) and another restricted to the relative abundance data for hopliine scarab beetles. Permutation tests comparing pollinator assemblages among flower colors were applied using ANOSIM implemented in PRIMER 6.1.15.

The relationship between geographical proximity of *D*. *cistiflora* s.l. floral color forms and the similarity of pollinator communities was assessed to determine whether pollinator assemblages were geographically structured and whether similarities in pollinator assemblages within or among floral color forms could reflect spatial proximity of populations. Pairwise distances between each population were calculated using ARCVIEW GIS 3.2, and a geographical distance matrix was produced. Mantel tests implemented in POPTOOLS (Hood, 1994) were used to assess the relationship between pairwise geographical distance and the Bray-Curtis index of the pollinator community composition of each population pair. Three separate analyses were performed, using data for: i) all populations, ii) populations with different flower colors, and iii) populations with the same flower color.

## Results

### Geographical Distribution of Floral Color Forms

**FIGURE 1:**
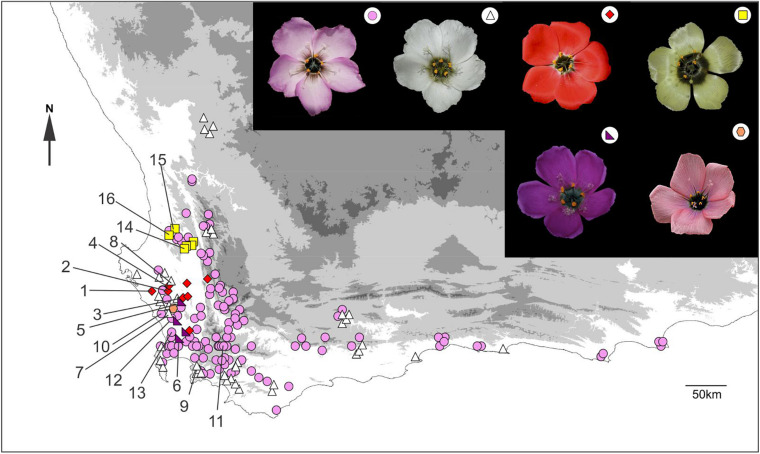
The geographical distribution of all known extant and extinct populations of *Drosera cistiflora* s.l. where corolla color data has been recorded. Recorded flower colors (see symbols) in decreasing order of frequency are pink (circles), white (triangles), red (diamonds), yellow (squares), purple (right-angled triangles), and salmon pink (hexagons). Populations studied (Supplementary Table S1) are labeled numerically. Photo of salmon pink form by Rob Maharajh.

Locality data were found for 168 *Drosera cistiflora* s.l. populations of known flower color in South Africa (Figure 1). The range of these sites spanned 685 km from west to east and 390 km from north to south. The majority of sites comprised either pink (108 sites: 64.3% of all sites) or white (41 sites: 24.4%) floral color forms. These two forms are widespread throughout the entire range of the species complex. Populations designated here as pink may show a continuum spanning pink to white, but populations designated as white consisted only of white-flowered individuals. Small clusters of three additional floral color forms are found in the central part of the range (Figure 1). Six yellow-flowered populations (3.6% of total sites) were found within 40 km of each other. To the south of these, nine red-flowered populations (5.3% of total sites) occurred within 83 km of each other, and further south, four purple-flowered populations (2.4% of total sites) were found within 38.5 km of each other. Two of the red-flowered populations co-occurred with purple-flowered individuals and another one co-occurred with white-flowered individuals. Purple-flowered populations also co-occurred with pink- or white-flowered populations at two sites. Apart from pink and white forms which sometimes showed a continuum, the different flower colors appeared to be discrete with no intermediates observed in zones where the distribution ranges of different forms overlapped.

**FIGURE 2:**
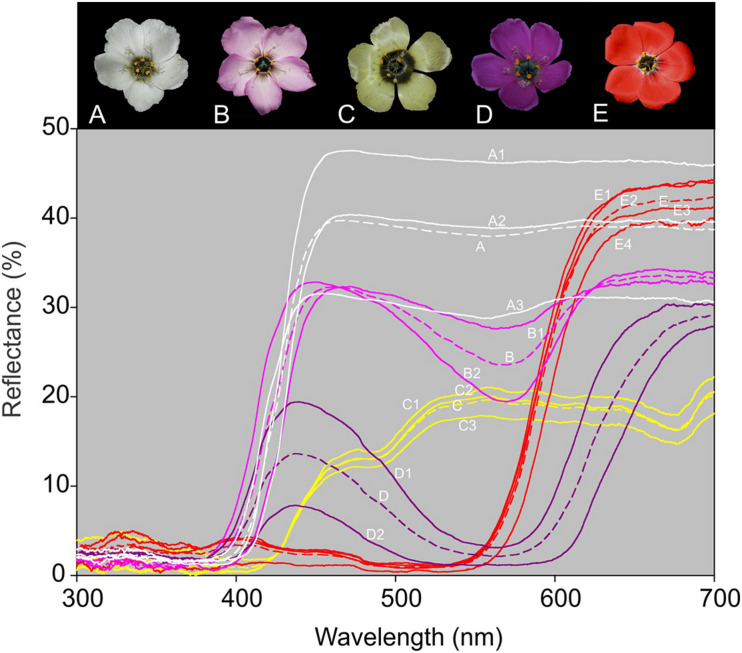
Spectrophotometer readings over the UV–visible range (300–700 nm) for the outer region of the petals of all five *Drosera cistiflora* s.l. floral color forms. Readings were obtained from 5 to 16 flowers from two populations of each of the pink **(B)** and purple **(D)**; three populations of white **(A)** and yellow **(C)**, and four of red **(E)** *D*. *cistiflora* s.l. floral color forms. Populations sampled comprised: Darling 5 (A1), Darling 4 (A2), and Betty’s Bay (A3) [white-flowered form]; Darling 7 (B1) and Darling 6 (B2) [pink-flowered form]; Piketberg 1 (C1), Piketberg 2 (C2), and Piketberg 3 (C3) [yellow-flowered form]; Darling 2 (D1) and Durbanville (D2) [purple-flowered form], and Darling 2 (E1), Darling 1 (E2), Darling 3 (E3), and Darling-Yzerfontein (E4) [red-flowered form]. Average readings for each floral color form are distinguished by dashed lines.

Measurements of reflectance spectra showed that UV light is not reflected by the upper petal surfaces of any of the floral color forms. Spectra of pink and white forms were not readily distinguishable, but spectra of red, yellow, and purple forms were discrete (Figure 2).

### Soil Types and Vegetation

Populations of *D*. *cistiflora* s.l. occur on at least 21 soil types. No floral color forms were edaphic endemics as all occurred on two or more soil types. Pink- and white-flowered forms were found on the most diverse range of soil types, reflecting the much larger distribution range of these two floral color forms. There was also considerable overlap of soil types between populations with different flower colors. For example, pink, purple, red, and white flowers could all be found on loam soils and pink, purple and white flowers could all be found on sandy soils (Supplementary Tables S2, S3).

No change in floral color expression was apparent for any of the 90 potted plants (18 per color form) with switched substrates and altered environmental conditions when they were examined in 2010, 2012, and 2013.

Each *D*. *cistiflora* s.l. floral color form was found in more than one vegetation type and most of these vegetation types supported more than one floral color form (Supplementary Table S4). For example, Atlantis Sand Fynbos and Swartland Granite Renosterveld support pink, purple, red and white floral color forms; Swartland Shale Renosterveld supports pink, purple and yellow floral color forms, and both red- and white-flowered forms also occur in Hopefield Sand Fynbos. The distribution of *D*. *cistiflora* s.l. flower colors was therefore not tightly linked with any specific plant communities.

### Pollinator Assemblages

We recorded a total of 1,169 individual insects as visitors to flowers of *D. cistiflora* s.l. (Supplementary Table S5). A total of 27 insect pollinator species from 11 families were observed in *D*. *cistiflora* s.l. flowers in 2009 and 2010 (summary in Table 1, details in Supplementary Table S5). The overwhelming majority (74%) of insect species recorded were beetles and most (44%) of these were hopliine scarabs (Table 1, Supplementary Table S5).

**TABLE 1:** Relative importance (RPI) values (%) of insect pollinators observed visiting each floral color form of *Drosera cistiflora* s.l. in 2009 and 2010.

		*Drosera cistiflora* s.l. floral color form				
Order and family	Species	Pink	Purple	Red	White	Yellow
Coleoptera						
Scarabaeidae: Hopliini	*Anisochelus inornatus*					16.5
	*Anisonyx* sp.				4.4	
	*Anisonyx cf*. *ursus*	2.3	2.0		0.19	
	*Chasme decora*			10.6		
	*Chasme* sp.			0.07		
	*Heterochelus* sp.					1.2
	*Lepisia rupicola*	29.6	15.2	**88.7**	**87.1**	
	*Lepithrix* sp.					**46.8**
	*Omocrates* sp.		**63.2**			
	*Peritrichia* sp. 1					6.2
	*Peritrichia* sp. 2					0.99
	*Platychelus lupinus*			0.28		
Scarabaeidae	sp. 1	1.3				0.28
Chrysomelidae	sp. 1	0.3		0.01	0.02	
Meloidae	sp. 1	**50.5**			4.5	
Melyridae	sp. 1	0.05				
	sp. 2	1.02			0.81	21.8
	sp. 3	13.4	19.2		2.5	2.6
Tenebrionidae	sp. 1			0.02		
	sp. 2	0.66			0.02	
Hymenoptera						
Megachilidae	sp. 1	0.50			0.32	
Diptera						
Muscidae	sp. 1	0.23				
Tabanidae	sp. 1			0.27		
	sp. 2					3.4
Ceratopogonidae	sp. 1	0.03	0.09	0.002	0.04	0.054
Empididae	sp. 1	0.04				
Hemiptera						
Lyganidae	sp. 1				0.121	

*Pollinator importance was calculated as the product of abundance in D. cistiflora s.l. flowers and average D. cistiflora s.l. pollen loads (Supplementary Table S5). Relative importance was calculated as the percentage contribution of each pollinator to the overall pollinator importance in each D. cistiflora s.l. floral color form. The pollinator group with the highest RPI value for each floral color form is indicated in bold type.*

**FIGURE 3:**
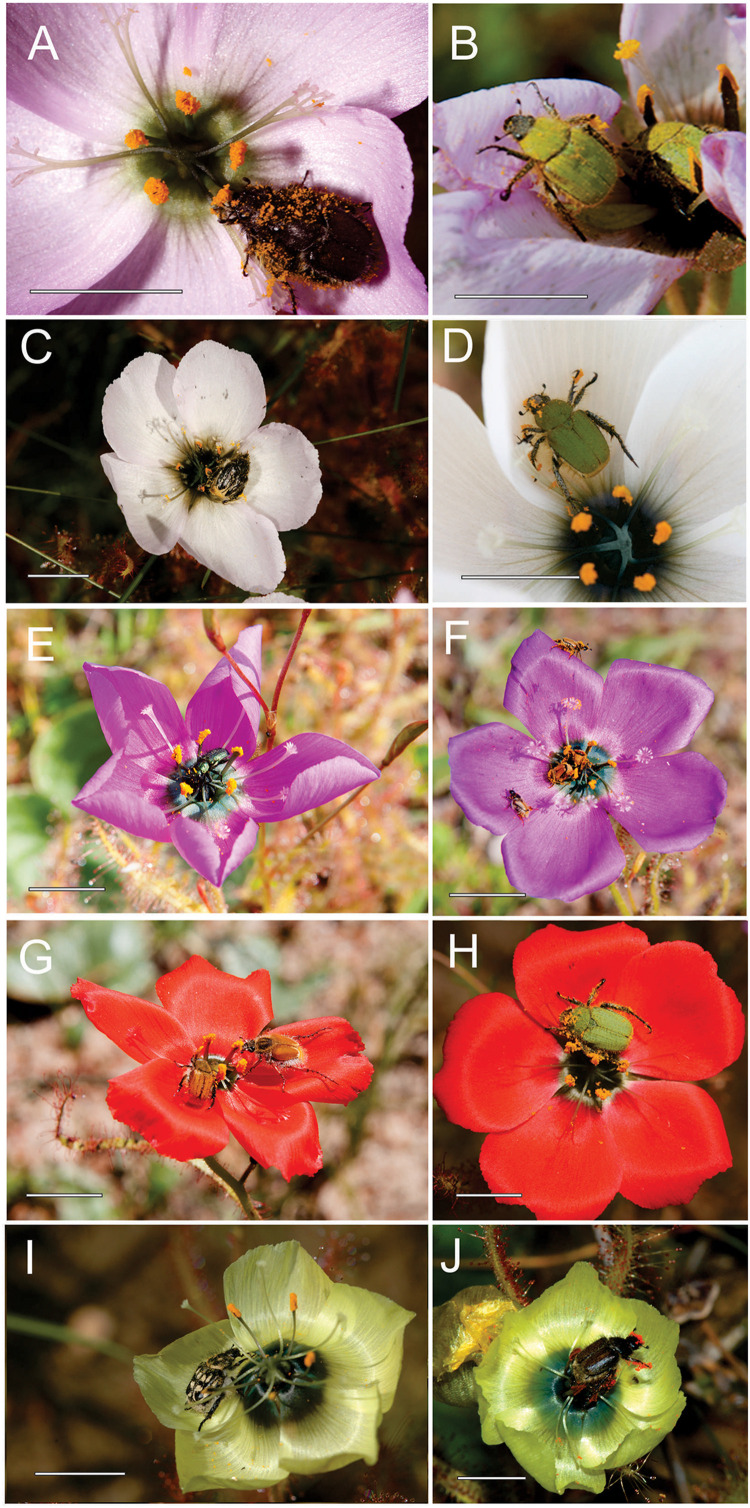
Hopliine beetle (Coleoptera: Scarabaeidae: Hopliini) **(A–D,F–J)** and melyrid beetle (Coleoptera: Melyridae) **(E)** pollinators on the five floral color forms of *Drosera cistiflora* s.l. *Lepisia rupicola* spec. **(B,D,H)** emerged as an important pollinator of pink- **(B)**, white- **(D)** and red-flowered **(H)** forms. *Omocrates* sp. **(F)** was abundant in purple-flowered forms and *Chasme decora* (**G**, left) was only observed on red flowers. *Peritrichia* sp. **(I)** and *Heterochelus* sp. **(J)** are shown visiting yellow flowers. Photo **(J)** by Kim Steiner. Scale bars = 10 mm.

Hopliine beetles (Coleoptera: Scarabaeidae: Hopliini) were evidently the primary pollinators of purple, red, white and yellow *D*. *cistiflora* s.l. floral color forms (Table 1, Figure 3, and Supplementary Table S5), with relative importance (RPI) per floral color form in descending order of magnitude as follows: red (99.7%), white (91.7%), purple (80.4%), yellow (71.8%), and pink (31.8%). Species assemblages of hopliine beetles differed largely between colors. Non-florivorous beetles of the family Meloidae were also important pollinators of pink-flowered forms (RPI: 50.5%), and soft-winged flower beetles (Melyridae) were of importance in pink-, purple-, and yellow-flowered forms, with RPI of 13.4, 19.2, and 21.8%, respectively. A florivorous lunate blister beetle *Hycleus lunatus* (Coleoptera: Meloidae: Meloinae: Mylabrini) was observed to consume flower parts (Supplementary Figure S2) and was excluded from the lists of potential pollinators. A species of hopliine beetle, *Lepisia rupicola*, had particularly high relative importance in red- (RPI: 88.7%), white- (RPI: 87.1%), and pink-flowered (RPI: 29.6%) populations, but was absent from yellow-flowered populations (Table 1, Supplementary Table S5). All *D*. *cistiflora* s.l. insect visitors were found to be polylectic (viz. not specific to *D*. *cistiflora* s.l.) and carried pollen from other plant species in the local environment.

**FIGURE 4:**
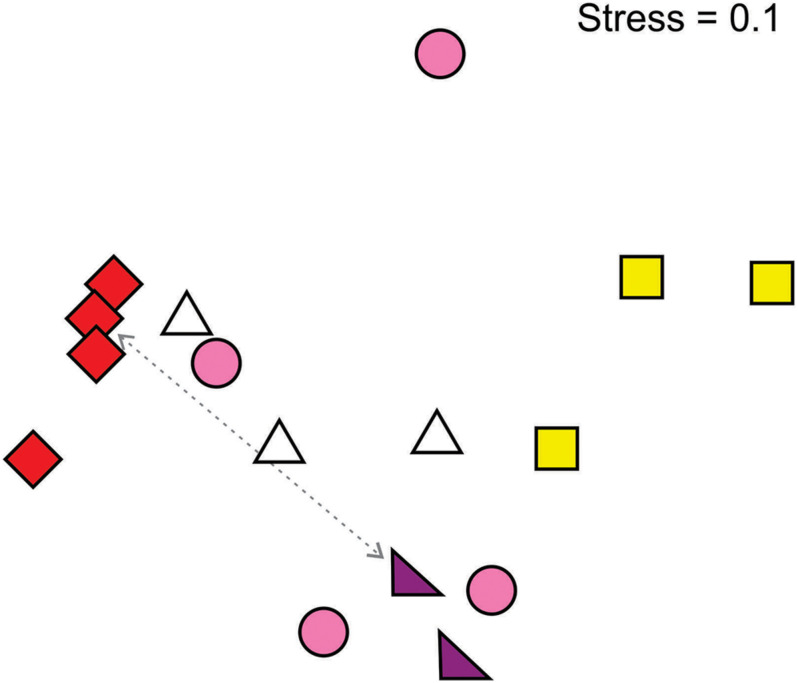
Multidimensional scaling (Bray-Curtis similarity index) plot of pollinator assemblages according to the flower color of *Drosera cistiflora* s.l. populations. Populations that are close together share similar pollinator communities while those that are far apart have different pollinator communities. Symbols differentiate *D*. *cistiflora* s.l. flower colors (see Figure 1 for a key). Two populations that are sympatric are indicated by a dotted line.

There was an overall relationship between flower color and pollinator community (Figure 4). In particular, red- and yellow-flowered populations formed distinct clusters on the basis of pollinator composition. Red-flowered populations clustered strongly and had a significantly different pollinating fauna to yellow- (*R* = 1.00, *p* = 0.03) and pink-flowered populations (*R* = 0.54, *p* = 0.03) and a marginally non-significant difference to white-flowered populations (*R* = 0.39, *p* = 0.06), while the fauna in yellow-flowered populations was significantly different to that in pink-flowered populations (*R* = 0.46, *p* = 0.03). Populations of pink and white floral color forms had more variable pollinator compositions and did not form discrete clusters. Given that there were only two purple-flowered populations, there was not enough statistical power to compare this form to the others.

Clustering weighted by relative pollinator importance did not strongly alter the associations between pollinator assemblage and flower color (global *R* = 0.48, *p* = 0.02, stress value = 0.09), so that red- and yellow-flowered populations still formed discrete groups (Supplementary Figure S3). Yellow-flowered populations were significantly different from those of red- (*R* = 1.00, *p* = 0.03) and pink-flowered (*R* = 0.51, *p* = 0.03) populations. The difference between red-flowered and pink-flowered populations was marginally non-significant (*R* = 0.46, *p* = 0.057) and all other pairwise combinations were non-significant.

In the NMDS analysis restricted to hopliine scarab beetle assemblages, there was again strong clustering of red- and yellow-flowered populations (Supplementary Figure S4), but red-flowered populations were not clearly separable from white- and pink-flowered populations and the only significant differences were between red- and yellow-flowered populations (*R* = 1, *P* = 0.029) and between yellow- and pink-flowered populations (*R* = 0.52, *P* = 0.029).

**FIGURE 5:**
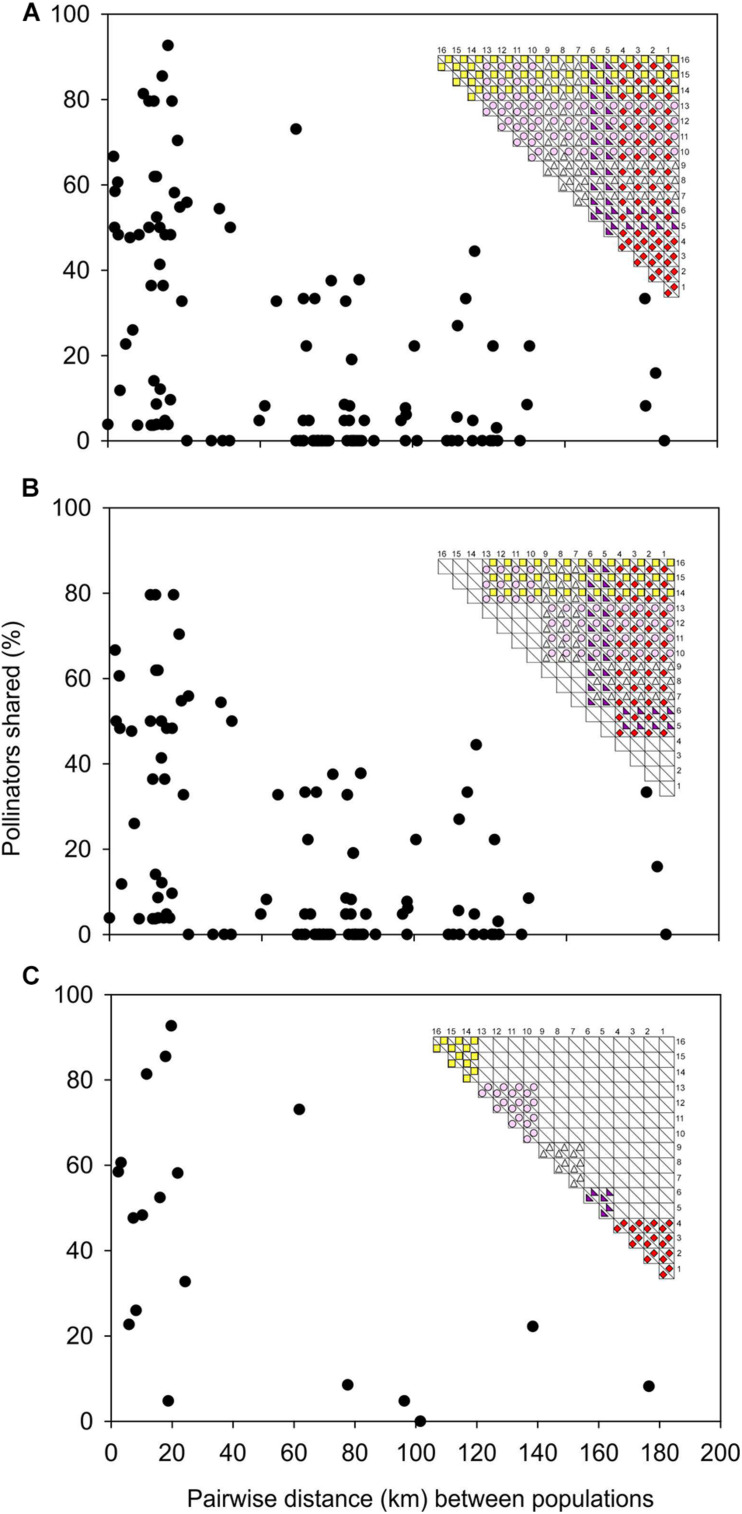
Relations between pairwise geographical distances and pairwise Bray-Curtis similarities of flower visitor assemblages in *Drosera cistiflora* s.l. populations, for all populations **(A)**, populations with different flower colors only **(B)**, and populations with the same flower color only **(C)**. Inset matrices show populations 1–16 and flower colors for which correlations are plotted. Each dot represents a population pair.

There was a significant negative relationship between pairwise geographical proximity of *D*. *cistiflora* s.l. populations and the pairwise similarity of the pollinating fauna *r*_*m*_ = −0.474, *P* < 0.0001 (Figure 5A), indicating that nearby populations shared similar pollinators while geographically distant populations had more dissimilar pollinating fauna compositions. This was also the case for pairs of populations with different flower colors (*r*_*m*_ = −0.417, *P* < 0.0001, Figure 5B) and those with the same flower color (*r*_*m*_ = −0.547, *P* < 0.0001, Figure 5C).

## Discussion

Floral color variation in the *D. cistiflora* complex is associated with switches in beetle pollinator assemblages dominated by hopliine scarab beetles. It is particularly notable that hopliine scarabs, along with glaphyrid scarabs in the Mediterranean, are among the few insect groups to include species that are strongly attracted to red flowers (Picker and Midgley, 1996; Johnson and Midgley, 2001; Van Kleunen et al., 2007; Streinzer et al., 2019). However, unlike glaphyrid scarabs that show consistent preferences for red and do not seem to clearly discriminate other colors, such as white, from the background foliage (Martinez-Harms et al., 2012; Streinzer et al., 2019), hopliine scarabs show a wide range of color preferences that vary markedly among individual species (Picker and Midgley, 1996; Steiner, 1998b; Johnson and Midgley, 2001; Van Kleunen et al., 2007; von Witt, 2019). Hopliine scarabs are thought to be responsible for a syndrome of dark-centered and bowl-shaped flowers with colors ranging from white through to red that have evolved in many different lineages in the Cape Floristic Region (Goldblatt et al., 1998; Van Kleunen et al., 2007). *Drosera cistiflora* provides an unusual case of a species complex where the divergence among floral color forms appears to have been driven by the foraging preferences of hopliine scarabs (von Witt, 2019).

We found no evidence suggesting that underlying soil or habitat type is associated with the color of *D. cistiflora* s.l. flowers. In fact, several sites (Supplementary Tables S1, S2) have more than one floral color form, occasionally growing intermingled. It is also clear that the natural distribution of each floral color form transcends many different soil types. In view of the absence of edaphic and vegetation type endemism in all *D*. *cistiflora* s.l. floral color forms, and given that vegetation types may serve as a proxy for multiple abiotic factors such as soil chemistry, temperature, light and moisture availability, flower color in *D. cistiflora* s.l. does not appear to be a manifestation of physiological responses to components of the physical environment. Furthermore, individuals transplanted across soil types also retain their original color over many flowering seasons, suggesting that color variation is not a phenotypically plastic trait. The transplant experiment does not provide conclusive evidence for this on its own, as the plants were not grown from seed (germination of the seeds is extremely difficult to achieve in cultivation). However, when seen in the context of co-existence of some color forms and a lack of overall association between soils and color, the evidence indicates that flower color is not simply a plastic response to soil chemistry.

Flower color in the genus *Drosera* globally and in South Africa is generally pink or white and this is also the case for most populations in the *D. cistiflora* complex (von Witt, 2019). The relatively terminal position of the species complex in the phylogeny of *Drosera* (Rivadavia et al., 2003) suggests that novel colors in the complex, such as red and yellow, are more recently derived modifications. The red- and yellow-flowered forms are specialized for pollination by hopliine scarab beetles and are also strongly diverged from one another in terms of their pollination niches. We suspect that these represent the evolution of novel pigment pathways (rather than loss of function transitions which tend to occur much more commonly – Rausher, 2008). Transitions involving loss of function can easily occur under very weak natural selection, or can even occur as a result of genetic drift when selection on color is weak or absent. However, it can be expected that the evolution of novel pigment pathways would have to occur under conditions of very strong directional selection. In this case the independent evolution of red and yellow colors in numerous other unrelated South African angiosperms pollinated by hopliine beetles (Steiner et al., 1987; Goldblatt et al., 1998; Johnson et al., 2004) provides a pattern of convergent evolution which is strongly suggestive of adaptive function. Another convergent feature found in c. 75% of plant species pollinated by hopliine scarabs is a dark floral center (Goldblatt et al., 1998; Johnson and Midgley, 2001). It has been suggested that these dark centers could represent mimicry of potential mating partners (Steiner, 1998a). There is evidence that dark floral centers increase the frequency of landings by hopliine beetles (Van Kleunen et al., 2007), but the strongest effect on their alighting behavior is the overall flower color (Johnson and Midgley, 2001). We frequently observed mating of hopliine scarabs on flowers of *D. cistiflora*, s.l. but the color of the dark center of the flowers does not usually correspond closely to the elytra of the beetles, which range in color from light green through to dark brown-black across species (Figure 3).

Closely related populations are expected to share traits through common descent and should also be geographically close to one another. We detected strong associations between geographic distance and pollinator community composition, suggesting that geographical proximity of similar color forms alone may explain associations between flower color and pollinator community composition. However, if geography was the only explanation for pollinator assemblages, different color forms within a site would be expected to have the same pollinator assemblages. This is not the case because at sites with more than one flower color we find strong pollinator partitioning among color forms. For example, the Darling 2 site has co-flowering purple and red forms, where the pollinator assemblage of the red form is more similar to that of relatively geographically distant red populations than the co-occurring purple-flowered plants (Figure 4). At this site, two hopliine beetle pollinators (*Lepisia rupicola* and *Chasme decora*) made up more than 95% of all insect visits to red flowers but made up only 3.8% of visits to purple flowers. In contrast, the most important visitors to purple flowers at this site were the hopliine beetle *Omocrates* sp. and melyrid beetles which were never observed on the red flowers. This pollinator partitioning also suggests a potentially strong role played by pollinators in reproductive isolation of emerging lineages (Liu and Huang, 2013). All *D*. *cistiflora* s.l. floral color forms, whether they occurred together at the same site, or apart at different sites, had overlapping flowering phenologies. However, while this suggests that pollinators select among flowers on the basis of color, it does not demonstrate that geographical color variation is locally adaptive. We have obtained evidence for local adaptation in a separate study in which arrays of different color forms of *D. cistiflora* s.l. were presented at each site (von Witt, 2019). This confirmed that there is strong discrimination by beetles among color forms and an overall preference of beetles for the local color forms.

A fundamental assumption of the Grant-Stebbins model of pollinator-driven diversification is that pollinator communities differ geographically (Grant and Grant, 1965; Stebbins, 1970; Johnson, 2006, 2010; Van der Niet et al., 2014). If the most effective pollinators in these different communities differ in their flower color preferences, we would expect a geographical selection mosaic that would result in the evolution of geographical differences in color forms. Since we sampled pollinators only on *Drosera* flowers, we have no independent assessment of pollinator distributions and the museum records of these insects are too sparse to create reliable distribution maps. This is a very common problem in studies that attempt to test the Grant-Stebbins model of diversification driven by geographical mosaics of pollinator availability (Johnson, 2006). However, plots of pollinator sharing by populations in relation to geographical distance (Figure 5) show that there is some degree of geographical structuring of pollinator assemblages that is independent of floral color forms. While this is not conclusive evidence for ultimate geographical structure in pollinator availability (as opposed to the proximate effects of floral traits on pollinator assemblages), it is consistent with one of the predictions of the Grant-Stebbins model.

Floral color shifts in *D*. *cistiflora* s.l. appear to represent adaptations to entire community compositions of pollinators and not specific species, since pollinator assemblages differed overall but also had many overlapping components. This would be consistent with the assertion of Gómez et al. (2008, 2014) that floral phenotypes of generalist plants may diversify across the range of a species in response to suites of pollinators (many of which may also be generalist). Similarly, findings of generalized pollination and spatial variation in visitor assemblage in *Calochortus* (Liliaceae) suggested that lineages may have been moving through a spatiotemporal mosaic of pollinators over evolutionary time (Dilley and Mesler, 2000). Here the authors surmised that color patterns, among other floral traits, have diverged through the historical accumulation of floral modifications that have been selected for by the suites of pollinators to which they appeal. Given that hopliine scarabs display highly variable color preferences among species (Picker and Midgley, 1996; Steiner, 1998b; Johnson and Midgley, 2001; Van Kleunen et al., 2007), spatiotemporal variation in pollinator assemblages that include hopliine beetles is a highly plausible explanation for the evolution of the floral color polymorphism in *D. cistiflora* s.l.

Although floral color forms of *D. cistiflora* s.l. clearly transcend soil and habitat types, we could expect steep environmental gradients to shape pollinator assemblages if insects use specific soils as nesting sites, or use soil-specific larval host plants. Indeed, this appears to be the case for hopliine beetles, which display remarkably high levels of species turnover across very short geographic distances, often corresponding to changes in vegetation and soil (Colville et al., 2002; Colville, 2009). As a result, many hopliine beetles are endemic to very narrow habitats (Colville, 2009). Thus the ultimate reasons for geographical structure in assemblages of these beetles may relate to their general habitat requirements. Indeed, shifts in pollination systems have frequently been associated with parallel shifts in soil types (Patterson and Givnish, 2004; Goldblatt and Manning, 2006; Van der Niet et al., 2006), and it is plausible that future examination of *D*. *cistiflora* s.l. pollinator biogeography may actually find pollinator assemblages to be determined in part by edaphic and/or other abiotic factors. As experiments show that beetle pollinators of *D. cistiflora* s.l. discriminate strongly among the color forms (von Witt, 2019), there is potential for floral colour divergence to proceed as “consequent radiation” (sensu Patterson and Givnish, 2004) via an indirect association of plants with the soils and/or other physical components of the environment supporting their pollinators.

## Conclusion

Our results show a pattern linking pollinator communities and flower color, and as such the most compelling explanation for floral color divergence in *D*. *cistiflora* s.l. is that it has been pollinator-driven. Hopliine beetles show strong color preferences when selecting flowers (Johnson and Midgley, 2001; Van Kleunen et al., 2007; von Witt, 2019). Studies have revealed at least three photoreceptor types in certain hopliine beetle species (Arnold, 2010), and this is consistent with findings of local color preferences of pollinators in the *D*. *cistiflora* complex (von Witt, 2019).

In addition to pollinator color choice experiments, further work should include study of *D*. *cistiflora* s.l. pigment biosynthetic pathways and their pleiotropic potential, and pleiotropic effects of non-pollinator biotic agents such as herbivores, pollen thieves and seed predators. Detailed molecular studies of *D. cistiflora* s.l. plant populations may ultimately isolate genetic differences between floral color forms and determine whether these are associated with pollinator shifts.

## Data Availability Statement

The original contributions presented in the study are included in the article/Supplementary Material; further inquiries can be directed to the corresponding author.

## Author Contributions

All authors participated in the design of the study. SDJ and CvW contributed equally as joint first authors. This manuscript is based on a Ph.D. thesis by CvW who collected the data and wrote up the results under the supervision of SDJ and BA. SDJ led the process of preparing the manuscript for publication. All authors contributed to the article and approved the submitted version.

## Conflict of Interest

The authors declare that the research was conducted in the absence of any commercial or financial relationships that could be construed as a potential conflict of interest.
