# Supplementary material for: Floral Color Variation in Drosera cistiflora Is Associated With Switches in Beetle Pollinator Assemblages
Source: Front Plant Sci. 2020 Nov 17;11:606259. doi: 10.3389/fpls.2020.606259 (PMC7704453; doi:10.3389/fpls.2020.606259)
Supplement: Supplementary file 1 [file Data_Sheet_1.docx]

**Table S1.** Population number; site name and locality description; flower colour; respective Compton Herbarium and Kirstenbosch National Botanical Garden Collections Nursery voucher accession numbers (where available); GPS location, and number of *Drosera cistiflora* s.l. flowers studied in 2009 and 2010 in all 16 study populations. Precise locality information has been omitted owing to the sensitive nature of these populations and their vulnerability to overcollection.

| Site number | Site name and locality description | Flower colour | Voucher accession number | Latitude | Longitude | Number of plants observed flowering in 2009 | Number of plants observed flowering in 2010 |
| --- | --- | --- | --- | --- | --- | --- | --- |
| 1 | Darling-Yzerfontein | Red |  | -33.3 | 18.3 | 55 | 50 |
| 2 | Darling 1 | Red | 1483806 | -33.2 | 18.3 | > 250 | > 250 |
| 3 | Darling 2 | Red | 1483801 | -33.3 | 18.5 | 210 | > 250 |
| 4 | Darling 3 | Red | 1483805 and 2019/2 | -33.2 | 18.3 | > 250 | > 250 |
| 5 | Darling 2 | Purple | 1483816 and 2019/1 | -33.3 | 18.5 | 190 | 179 |
| 6 | Durbanville | Purple | 1483810 | -33.8 | 18.6 | 38 | 24 |
| 7 | Darling 4 | White | 2019/6 | -33.4 | 18.4 | > 250 | > 250 |
| 8 | Darling 5 | White | 1483804 | -33.2 | 18.3 | > 250 | 204 |
| 9 | Betty’s Bay | White |  | -34.4 | 18.9 | N/a | 49 |
| 10 | Darling 6 | Pink | 1483809 | -33.4 | 18.4 | 222 | 205 |
| 11 | Rawsonville | Pink |  | -33.7 | 19.4 | 190 | N/a |
| 12 | Darling 7 | Pink | 1483807 and 2019/5 | -33.4 | 18.4 | > 250 | > 250 |
| 13 | Riverlands Nature Reserve | Pink | 1483811 | -33.5 | 18.6 | 187 | N/a |
| 14 | Piketberg 1 | Yellow | 1483812 and 2019/4 | -32.8 | 18.8 | > 250 | > 250 |
| 15 | Piketberg 2 | Yellow | 1483815 | -32.7 | 18.7 | > 250 | > 250 |
| 16 | Piketberg 3 | Yellow | 1483813 | -32.7 | 18.7 | 220 | 200 |

**Table S2.** Detailed geology and vegetation attributes of GPS-georeferenced populations of *Drosera cistiflora* s.l. floral colour forms. GPS points and other precise locality information have been omitted owing to the sensitive nature of these populations and their vulnerability to overcollection. Occurrence according to detailed vegetation types is as follows: pink-flowered forms occur in a minimum of 15 vegetation types, with the majority of plants found in Swartland Granite Renosterveld (20.5% of pink-flowered populations), Breede Shale Renosterveld (12.8%), Swartland Shale Renosterveld (12.8%) and Atlantis Sand Fynbos (10.3%); purple-flowered forms occur in Atlantis Sand Fynbos (50%), Swartland Granite Renosterveld (25%) and Swartland Shale Renosterveld (25%); red-flowered forms appear in Sand Fynbos (66.7% Hopefield Sand Fynbos and 16.7% Atlantis Sand Fynbos) and Swartland Granite Renosterveld (16.7%); white-flowered forms emerge in at least six vegetation types, including Swartland Granite Renosterveld (25%), Atlantis Sand Fynbos (25%), Hopefield Sand Fynbos (16.7%), Kogelberg Sandstone Fynbos (16.7%), Bokkeveld Sandstone Fynbos (8.3%) and Overberg Sandstone Fynbos (8.3%), and yellow-flowered forms only occur in Piketberg Sandstone Fynbos (66.7%) and Swartland Shale Renosterveld (33.3%).

| Colour form | Locality | Geology code and description | Vegetation type |
| --- | --- | --- | --- |
|  |  |  |  |
| Pink | Abbotsdale | NCa (Granite, mainly coarse-grained porphyritic with fine-grained leucocratic, fine-to-medium-grained porphyritic and medium-grained biotitic variants) | Swartland Granite Renosterveld |
| Pink | Bain's Kloof | Qt (Gritty sand; scree) | Hawequas Sandstone Fynbos |
| Pink | Between Worcester and Villiersdorp | Qs (Light-grey to pale-red sandy soil) | Breede Quartzite Fynbos |
| Pink | Caledon | Dr (Light-grey feldspathic sandstone, siltstone and micaceous shale bands) | Rûens Silcrete Renosterveld |
| Pink | Ceres 1 | Dv (Shale, siltstone and subordinate sandstone; fossiliferous) | Ceres Shale Renosterveld |
| Pink | Ceres 2 | Qt (Gritty sand; scree) | Breede Alluvium Fynbos |
| Pink | Darling 6 (i) | NCd (Granite, mainly coarse-grained porphyritic with porphyritic biotitic, leucocratic, oven-grained biotitic and tourmaline-bearing variants; granodiorite) | Swartland Granite Renosterveld |
| Pink | Darling 6 (ii) | NCd (Granite: mainly coarse-grained porphyritic with porphyritic biotitic, leucocratic, oven-grained biotitic and tourmaline-bearing variants; granodiorite) | Swartland Granite Renosterveld |
| Pink | Darling 7 (i) | NCd (Granite, mainly coarse-grained porphyritic with porphyritic biotitic, leucocratic, oven-grained biotitic and tourmaline-bearing variants; granodiorite) | Swartland Granite Renosterveld |
| Pink | Darling 7 (ii) | NCd (Granite, mainly coarse-grained porphyritic with porphyritic biotitic, leucocratic, oven-grained biotitic and tourmaline-bearing variants; granodiorite) | Swartland Granite Renosterveld |
| Pink | Darling 8 | Qgg (Gravelly clay/loam soil) | Swartland Granite Renosterveld |
| Pink | Durbanville 1 (i) | Qg (Loam and sandy loam) | Swartland Shale Renosterveld |
| Pink | Durbanville 1 (ii) | Nt (Greywacke, phyllite and quartzitic sandstone; interbedded lava and tuff) | Swartland Shale Renosterveld |
| Pink | Durbanville 2 | Qs (Light-grey to pale-red sandy soil) | Swartland Shale Renosterveld |
| Pink | Joostenberg | NCs (Granite, mainly coarse-grained porphyritic with medium-to-coarse-grained, fine-grained porphyritic, fine-grained leucocratic, hybridic, fine-to-medium-grained tourmaline-bearing and coarse-grained biotitic variants) | Swartland Granite Renosterveld |
| Pink | Karwyderskraal Road | Dv (Shale, siltstone and subordinate sandstone; fossiliferous) | Kogelberg Sandstone Fynbos |
| Pink | Napier | Dv (Shale, siltstone and subordinate sandstone; fossiliferous) | Western Rûens Shale Renosterveld |
| Pink | Paardeberg | Qs (Light-grey to pale-red sandy soil) | Swartland Granite Renosterveld |
| Pink | Philadelphia | Qf (Ferricrete) | Swartland Shale Renosterveld |
| Pink | Rawsonville | Sg (Red-brown-weathering, thin-bedded quartzitic sandstone, thin shale beds in places) | Hawequas Sandstone Fynbos |
| Pink | Riverlands Nature Reserve (i) | Qs (Light-grey to pale-red sandy soil) | Atlantis Sand Fynbos |
| Pink | Riverlands Nature Reserve (ii) | Qs (Light-grey to pale-red sandy soil) | Atlantis Sand Fynbos |
| Pink | Riverlands Nature Reserve (iii) | Qs (Light-grey to pale-red sandy soil) | Atlantis Sand Fynbos |
| Pink | Riverlands Nature Reserve (iv) | Qg (Loam and sandy loam) | Atlantis Sand Fynbos |
| Pink | Stellenbosch | NCs (Granite, mainly coarse-grained porphyritic with medium-to-coarse-grained, fine-grained porphyritic, fine-grained leucocratic, hybridic, fine-to-medium-grained tourmaline-bearing and coarse-grained biotitic variants) | Boland Granite Fynbos |
| Pink | Swellendam | Qg (Loam and sandy loam) | Eastern Rûens Shale Renosterveld |
| Pink | Swellendam | Dw (Micaceous siltstone, shale and subordinate sandstone) | Eastern Rûens Shale Renosterveld |
| Pink | Tulbagh 1 | Npo (Phyllite shale, schist and greywacke with dark-grey limestone, sporadic quartzitic sandstone beds and conglomerate beds) | Breede Shale Fynbos |
| Pink | Tulbagh 2 | Npo (Phyllite shale, schist and greywacke with dark-grey limestone, sporadic quartzitic sandstone beds and conglomerate beds) | Breede Shale Renosterveld |
| Pink | Tulbagh 3 | Npo (Phyllite shale, schist and greywacke with dark-grey limestone, sporadic quartzitic sandstone beds and conglomerate beds) | Breede Shale Fynbos |
| Pink | Tulbagh 4 | Npo (Phyllite shale, schist and greywacke with dark-grey limestone, sporadic quartzitic sandstone beds and conglomerate beds) | Breede Shale Renosterveld |
| Pink | Tulbagh 5 | Npo (Phyllite shale, schist and greywacke with dark-grey limestone, sporadic quartzitic sandstone beds and conglomerate beds) | Breede Shale Renosterveld |
| Pink | Tulbagh 6 | Npo (Phyllite shale, schist and greywacke with dark-grey limestone, sporadic quartzitic sandstone beds and conglomerate beds) | Breede Shale Renosterveld |
| Pink | Villiersdorp | Dr (Light-grey feldspathic sandstone, siltstone and micaceous shale bands) | Breede Shale Renosterveld |
| Pink | Wellington 1 | Nn (Phyllite, medium-grained to gritty greywacke, feldspathic and sericitic quartzite, limestone, dolomite and gritstone; greenstone, highly sheared and partly replaced by calcite and chert) | Swartland Shale Renosterveld |
| Pink | Wellington 2 | Qs (Light-grey to pale-red sandy soil) | Swartland Alluvium Fynbos |
| Pink | Wolseley (i) | Npo (Phyllite shale, schist and greywacke with dark-grey limestone, sporadic quartzitic sandstone beds and conglomerate beds) | Breede Alluvium Fynbos |
| Pink | Wolseley (ii) | Npo (Phyllite shale, schist and greywacke with dark-grey limestone, sporadic quartzitic sandstone beds and conglomerate beds) | Breede Alluvium Fynbos |
| Pink | Wolseley (iii) | Npo (Phyllite shale, schist and greywacke with dark-grey limestone, sporadic quartzitic sandstone beds and conglomerate beds) | Breede Shale Fynbos |
| Purple | Darling 2 | Qg (Loam and sandy loam) | Atlantis Sand Fynbos |
| Purple | Durbanville 2 | Qs (Light-grey to pale-red sandy soil) | Swartland Shale Renosterveld |
| Purple | Malmesbury District | Nm (Greywacke and phyllite with beds and lenses of quartz schist, limestone and grit; quartz-sericite schist with occasional limestone lenses) | Atlantis Sand Fynbos |
| Purple | Paarl | Nm (Greywacke and phyllite with beds and lenses of quartz schist, limestone and grit; quartz-sericite schist with occasional limestone lenses) | Swartland Granite Renosterveld |
| Red | Darling-Yzerfontein | NCd (Granite, mainly coarse-grained porphyritic with porphyritic biotitic, leucocratic, oven-grained biotitic and tourmaline-bearing variants; granodiorite) | Hopefield Sand Fynbos |
| Red | Darling 1 (i) | Qg (Loam and sandy loam) | Hopefield Sand Fynbos |
| Red | Darling 1 (ii) | Qg (Loam and sandy loam) | Hopefield Sand Fynbos |
| Red | Darling 2 | Qg (Loam and sandy loam) | Atlantis Sand Fynbos |
| Red | Hopefield | Qg (Loam and sandy loam) | Hopefield Sand Fynbos |
| Red | Paarl | Nm (Greywacke and phyllite with beds and lenses of quartz schist, limestone and grit; quartz-sericite schist with occasional limestone lenses) | Swartland Granite Renosterveld |
| White | Abbotsdale | Nca (Granite, mainly coarse-grained porphyritic with fine-grained leucocratic, fine-to-medium-grained porphyritic and medium-grained biotitic variants) | Swartland Granite Renosterveld |
| White | Betty's Bay | Os (Light-grey quartzitic sandstone with thin siltstone, shale and polymictic conglomerate beds) | Kogelberg Sandstone Fynbos |
| White | Darling 1 | Qg (Loam and sandy loam) | Hopefield Sand Fynbos |
| White | Darling 4 | NCd (Granite, mainly coarse-grained porphyritic with porphyritic biotitic, leucocratic, oven-grained biotitic and tourmaline-bearing variants; granodiorite) | Swartland Granite Renosterveld |
| White | Darling 5 | Qg (Loam and sandy loam) | Hopefield Sand Fynbos |
| White | Darling 9 | NCd (Granite, mainly coarse-grained porphyritic with porphyritic biotitic, leucocratic, oven-grained biotitic and tourmaline-bearing variants; granodiorite) | Atlantis Sand Fynbos |
|  |  |  |  |
| White | Hermanus | Ss (Light-grey, massively bedded, quartzitic sandstone; thin lenticular conglomerate and grit beds) | Kogelberg Sandstone Fynbos |
| White | Malmesbury District | Nm (Greywacke and phyllite with beds and lenses of quartz schist, limestone and grit; quartz-sericite schist with occasional limestone lenses) | Atlantis Sand Fynbos |
| White | Nieuwoudtville | Ss (Light-grey, massively bedded, quartzitic sandstone; thin lenticular conglomerate and grit beds) | Bokkeveld Sandstone Fynbos |
| White | Stanford | Qs (Light-grey to pale-red sandy soil) | Overberg Sandstone Fynbos |
| White | Yzerfontein | QI (Limestone and calcrete, partially cross-bedded, calcified parabolic dune sand) | Atlantis Sand Fynbos |
| Yellow | Piketberg 1 | Np (Grit and greywacke) | Swartland Shale Renosterveld |
| Yellow | Piketberg 2 | Og (Thinly-bedded sandstone, siltstone and mudstone, mainly reddish) | Piketberg Sandstone Fynbos |
| Yellow | Piketberg 3 | Np (Grit and greywacke) | Piketberg Sandstone Fynbos |

**Table S3**. Percentage occurrence of the five *Drosera cistiflora* s.l. floral colour forms on different soil types. Geology code descriptions are provided in Supplementary Table S2.

| Geology code | Occurrence (%) | | | | |
| --- | --- | --- | --- | --- | --- |
|  | Pink | Purple | Red | White | Yellow |
| Dr | 5.1 |  |  |  |  |
| Dv | 7.7 |  |  |  |  |
| Dw | 2.6 |  |  |  |  |
| NCa | 2.6 |  |  | 8.3 |  |
| NCd | 10.3 |  | 16.7 | 25.0 |  |
| NCs | 5.1 |  |  |  |  |
| Nm |  | 50.0 | 16.7 | 8.3 |  |
| Nn | 2.6 |  |  |  |  |
| Np |  |  |  |  | 66.7 |
| Npo | 23.1 |  |  |  |  |
| Nt | 2.6 |  |  |  |  |
| Og |  |  |  |  | 33.3 |
| Os |  |  |  | 8.3 |  |
| Qf | 2.6 |  |  |  |  |
| Qg | 7.7 | 25.0 | 66.7 | 16.7 |  |
| Qgg | 2.6 |  |  |  |  |
| QI |  |  |  | 8.3 |  |
| Qs | 18.0 | 25.0 |  | 8.3 |  |
| Qt | 5.1 |  |  |  |  |
| Sg | 2.6 |  |  |  |  |
| Ss |  |  |  | 16.7 |  |

**Table S4.** Percentage occurrence of the five *Drosera cistiflora* s.l. floral colour forms in broad vegetation categories. Specific vegetation types are provided in Supplementary Table S2.

| Broad vegetation category | Occurrence (%) | | | | |
| --- | --- | --- | --- | --- | --- |
|  | Pink | Purple | Red | White | Yellow |
| Alluvium fynbos | 10.3 |  |  |  |  |
| Granite fynbos | 2.6 |  |  |  |  |
| Granite renosterveld | 20.5 | 25.0 | 16.7 | 25.0 |  |
| Quartzite fynbos | 2.6 |  |  |  |  |
| Sand fynbos | 10.3 | 50.0 | 83.3 | 41.7 |  |
| Sandstone fynbos | 7.7 |  |  | 33.3 | 66.7 |
| Shale fynbos | 7.7 |  |  |  |  |
| Shale renosterveld | 35.9 | 25.0 |  |  | 33.3 |
| Silcrete renosterveld | 2.6 |  |  |  |  |

**Table S5.** Abundance, average pollen loads, and estimated importance of each insect pollinator found in *Drosera cistiflora* s.l. flowers during observations in 2009 and 2010. Superscript H denotes hopliine beetles (Coleoptera: Scarabaeidae: Hopliini).

| *Drosera cistiflora*s.l. floral colour form | Order and family | Species | Abundance |  | Average pollen load | Pollinator importance  (average pollen load × abundance) |
| --- | --- | --- | --- | --- | --- | --- |
| Pink | Coleoptera |  |  |  |  |  |
|  | Scarabaeidae^H^ | *Anisonyx cf*. *ursus* | 11 |  | 65.5 | 720.9 |
|  |  | *Lepisia rupicola* | 19 |  | 487.9 | 9270.5 |
|  | Scarabaeidae | sp. 1 | 15 |  | 28.0 | 420.0 |
|  | Tenebrionidae | sp. 2 | 69 |  | 3 | 207.0 |
|  | Meloidae | sp. 1 | 53 |  | 299.0 | 15847.0 |
|  | Melyridae | sp. 1 | 6 |  | 2.6 | 15.6 |
|  |  | sp. 2 | 3 |  | 106.6 | 320.0 |
|  |  | sp. 3 | 129 |  | 32.5 | 4188.6 |
|  | Chrysomelidae | sp. 1 | 39 |  | 2.5 | 98.3 |
|  | Diptera |  |  |  |  |  |
|  | Ceratopogonidae | sp. 1 | 14 |  | 0.8 | 10.5 |
|  | Empididae | sp. 1 | 5 |  | 2.5 | 12.5 |
|  | Muscidae | sp. 1 | 4 |  | 18.3 | 73.3 |
|  | Hymenoptera |  |  |  |  |  |
|  | Megachilidae | sp. 1 | 3 |  | 52.5 | 157.5 |
| Purple | Coleoptera |  |  |  |  |  |
|  | Scarabaeidae^H^ | *Anisonyx cf*. *ursus* | 1 |  | 65.5 | 65.5 |
|  |  | *Lepisia rupicola* | 1 |  | 487.9 | 487.9 |
|  |  | *Omocrates sp*. 1 | 14 |  | 145.0 | 2030.0 |
|  | Melyridae | sp. 3 | 19 |  | 32.5 | 616.9 |
|  | Diptera |  |  |  |  |  |
|  | Ceratopogonidae | sp. 1 | 4 |  | 0.8 | 3.0 |
| Red | Coleoptera Scarabaeidae^H^ | *Chasme decora* | 47 |  | 402.6 | 18921.3 |
|  |  | *Chasme* sp. 1 | 4 |  | 31.3 | 125.0 |
|  |  | *Lepisia rupicola* | 323 |  | 487.9 | 157598.2 |
|  |  | *Platychelus lupinus* | 7 |  | 73.3 | 513.3 |
|  | Chrysomelidae | sp. 1 | 5 |  | 2.5 | 12.6 |
|  | Tenebrionidae | sp. 1 | 20 |  | 1.8 | 35.0 |
|  | Diptera |  |  |  |  |  |
|  | Ceratopogonidae | sp. 1 | 4 |  | 0.8 | 3.0 |
|  | Tabanidae | sp. 1 | 3 |  | 163.3 | 490.0 |
|  |  |  |  |  |  |  |
| White | Coleoptera |  |  |  |  |  |
|  | Scarabaeidae^H^ | *Anisonyx* sp. 1 | 16 |  | 180.0 | 2880.0 |
|  |  | *Anisonyx cf*. *ursus* | 2 |  | 65.5 | 131.1 |
|  |  | *Lepisia rupicola* | 118 |  | 487.9 | 57574.6 |
|  | Chrysomelidae | sp. 1 | 5 |  | 2.5 | 12.6 |
|  | Meloidae | sp. 1 | 10 |  | 299.0 | 2990.0 |
|  | Melyridae | sp. 2 | 5 |  | 106.7 | 533.4 |
|  |  | sp. 3 | 50 |  | 32.5 | 1623.5 |
|  | Tenebrionidae | sp. 2 | 5 |  | 3.0 | 15.0 |
|  | Diptera |  |  |  |  |  |
|  | Ceratopogonidae | sp. 1 | 35 |  | 0.8 | 26.3 |
|  | Hymenoptera |  |  |  |  |  |
|  | Megachilidae | sp. 1 | 4 |  | 52.5 | 210.0 |
|  | Hemiptera |  |  |  |  |  |
|  | Lyganidae | sp. 1 | 4 |  | 20.0 | 80.0 |
| Yellow | Coleoptera |  |  |  |  |  |
|  | Scarabaeidae^H^ | *Anisochelus inornatus* | 20 |  | 80.8 | 1616.6 |
|  |  | *Heterochelus* sp. 1 | 1 |  | 120.0 | 120.0 |
|  |  | *Lepithrix* sp. 1 | 27 |  | 169.5 | 4575.7 |
|  |  | *Peritrichia* sp. 1 | 5 |  | 122.0 | 610.0 |
|  |  | *Peritrichia* sp. 2 | 1 |  | 96.9 | 96.9 |
|  | Scarabaeidae | sp. 1 | 1 |  | 28.0 | 28.0 |
|  | Melyridae | sp. 2 | 20 |  | 106.7 | 2133.6 |
|  |  | sp. 3 | 8 |  | 32.5 | 259.8 |
|  | Diptera |  |  |  |  |  |
|  | Ceratopogonidae | sp. 1 | 7 |  | 0.8 | 5.3 |
|  | Tabanidae | sp. 2 | 3 |  | 110.0 | 330 |


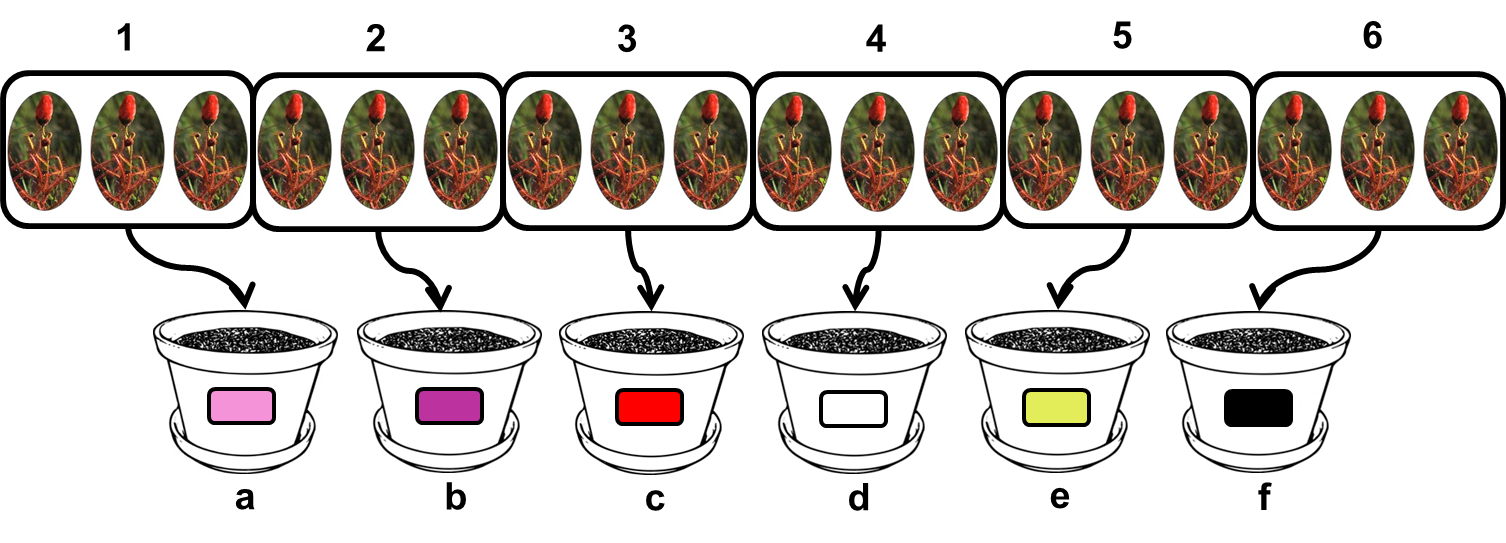


**Fig S1.** Experimental design of the common-garden and soil switching experiment, using the red *Drosera cistiflora* s.l. floral colour form from Darling 3 as an example. Separate samples of three plants in bud (1–6) were grown in soil from each *D*. *cistiflora* s.l. floral colour form (a–e) as well as from a site where the plants did not occur (f). Control plants (c) were potted in their native soils. The experiment was carried out using plants from one population of each of the pink-, purple-, red-, white- and yellow-flowered *D*. *cistiflora* s.l. forms. All experimental plants were kept in common environmental conditions and experienced common changes in temperature, light and moisture availability. Plants and soils were obtained from Darling 7 (granite and granodiorite soils supporting the pink-flowered form); Darling 2 (loam soils; purple-flowered form); Darling 3 (loam soils; red-flowered form); Darling 4 (granite and granodiorite soils; white-flowered form), and Piketberg 1 (grit and greywacke soils; yellow-flowered form). Clay soils collected from The Towers Farm, Darling were used for potting plants in soil from a site where *D*. *cistiflora* s.l. did not occur.


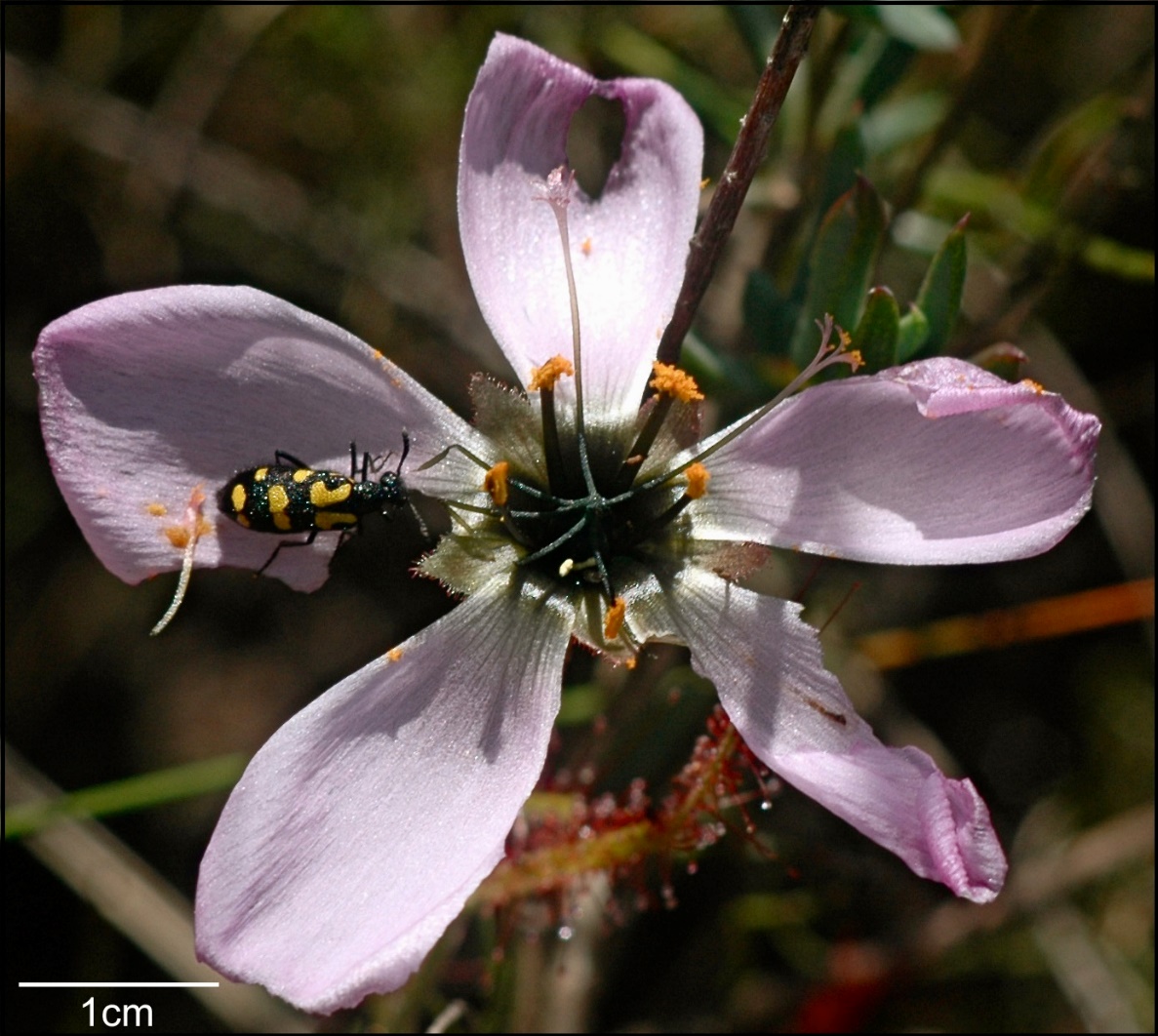


**Fig S2.** Floral destruction by the lunate blister beetle *Hycleus lunatus* (Coleoptera: Meloidae: Meloinae: Mylabrini), the only florivore observed visiting *Drosera cistiflora* s.l.


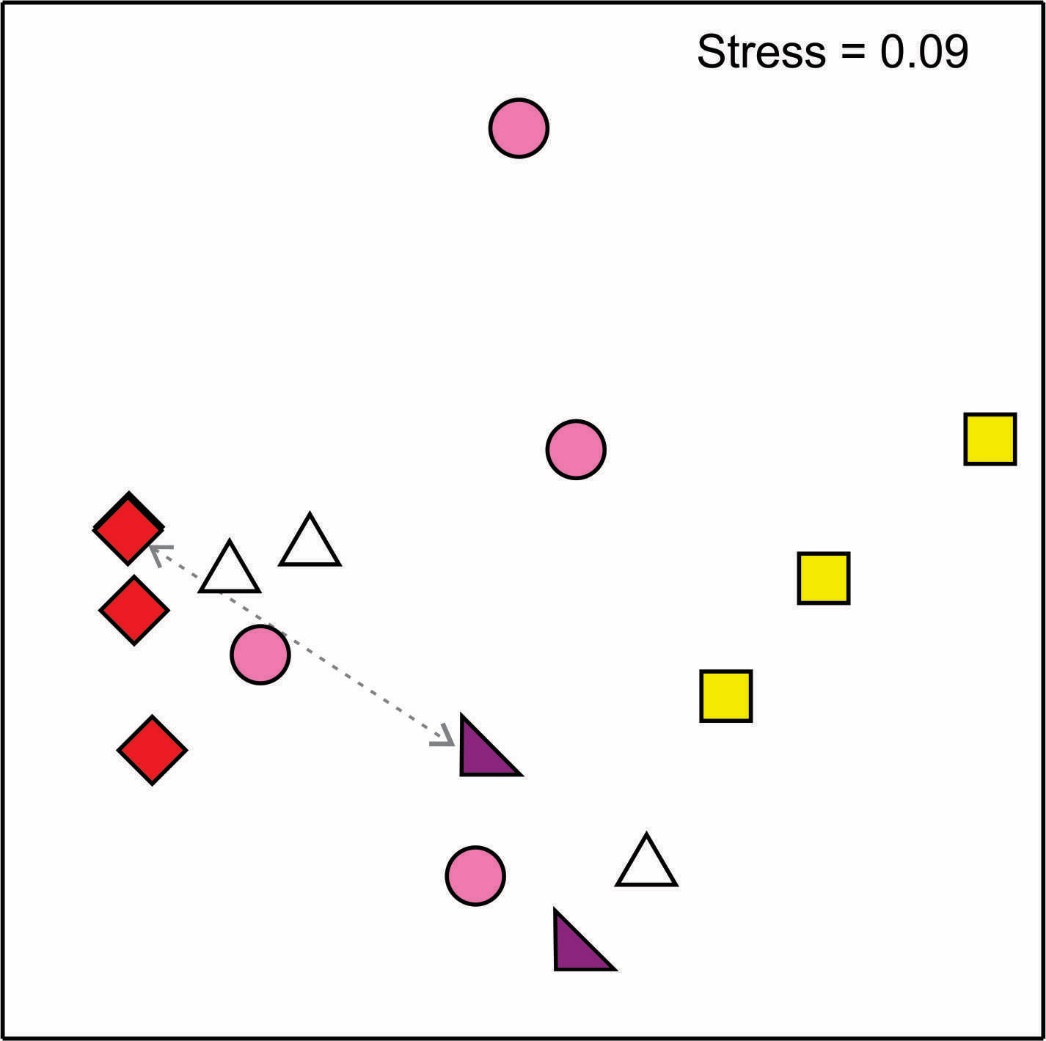


**Supplementary Figure S3.** Multidimensional scaling (Bray-Curtis similarity index) plot of pollinator assemblages weighted by pollinator effectiveness and plotted according to the flower colour of *Drosera cistiflora* s.l. populations. Populations that are close together share similar pollinator communities whilst those that are far apart have different pollinator communities. Symbols differentiate *D*. *cistiflora* s.l. flower colours (see Fig 1 for a key). Sympatric populations are indicated by a dotted line.


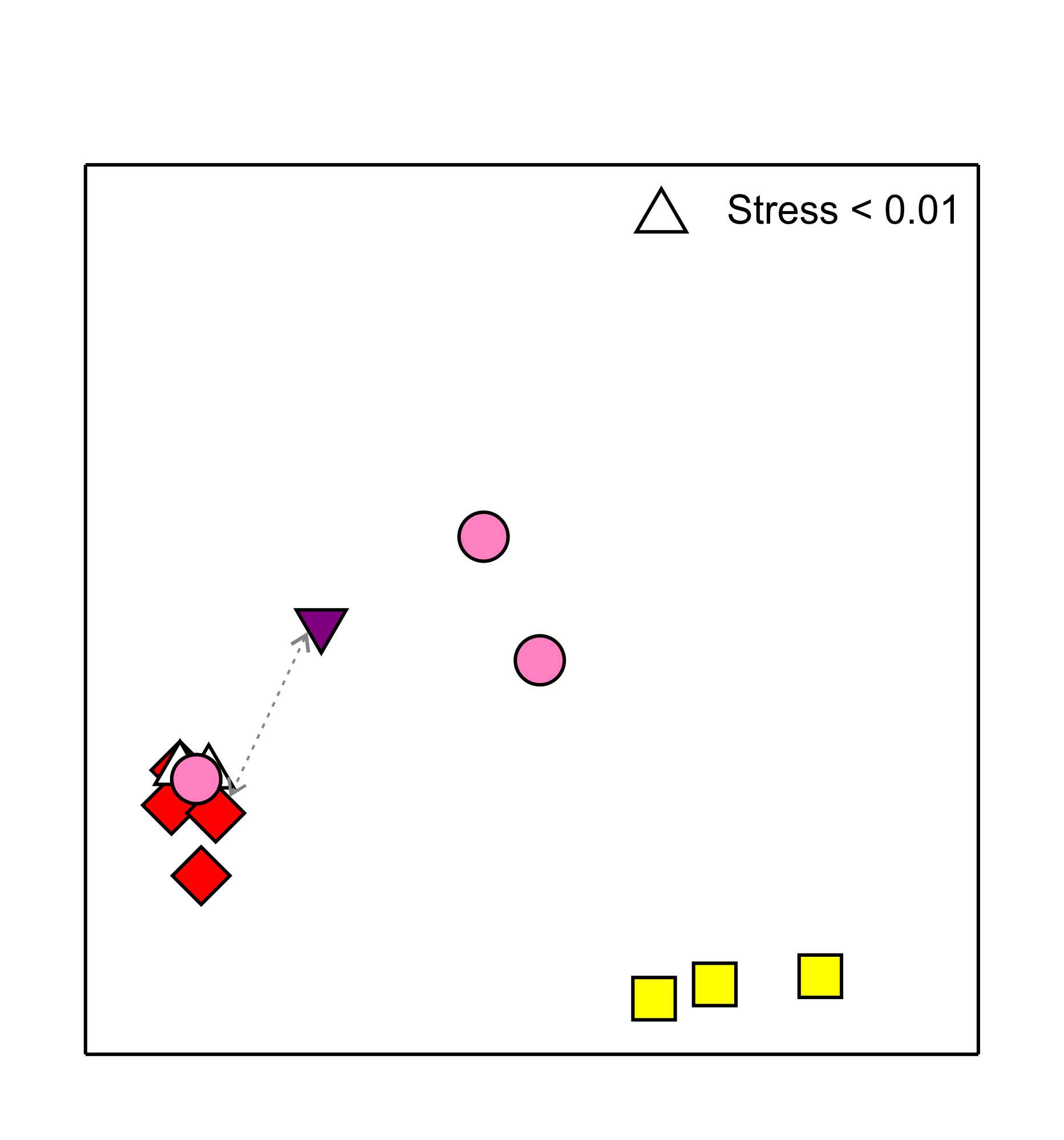


**Supplementary Figure S4.** Multidimensional scaling (Bray-Curtis similarity index) plot of hopliine scarab assemblages plotted according to the flower colour of *Drosera cistiflora* s.l. populations. Populations that are close together share similar pollinator communities whilst those that are far apart have different pollinator communities. Symbols differentiate *D*. *cistiflora* s.l. flower colours (see Fig 1 for a key). Sympatric populations are indicated by a dotted line
